# Epigenetics of Hypogonadotropic Hypogonadism: Molecular Mimicry between Severe Acute Respiratory Syndrome Coronavirus 2 and KISSR

**DOI:** 10.1055/s-0043-1770767

**Published:** 2023-06-23

**Authors:** Darja Kanduc

**Affiliations:** 1 Department of Biosciences, Biotechnologies and Biopharmaceutics, University of Bari, Bari, Italy

**Keywords:** KISSR, SARS-CoV-2, autoimmunity: hypogonadotropic hypogonadism

## Abstract

This study analyzed KISS1 and its receptor KISSR for peptide sharing with severe acute respiratory syndrome coronavirus 2 (SARS-CoV-2). It was found that SARS-CoV-2 shares numerous minimal immune pentapeptide determinants with KISSR only. The peptide sharing has a high immunologic potential since almost all the common peptides are present in 101 SARS-CoV-2-derived immunoreactive epitopes. Data are in favor of configuring molecular mimicry as an epigenetic factor that can alter KISSR thus causing the hypogonadotropic hypogonadism syndrome with which altered KISSR associates.

## Introduction

The human kisspeptin protein (here referred to as KISS1) and the human kisspeptin receptor protein (here referred to as KISSR) form the hypothalamic system that regulates the gonadotropic axis at puberty and in adulthood. 1 As reviewed by Szydełko-Gorzkowicz et al, 2 KISS1 and KISSR participate in different biological processes in that KISS1 plays an essential role in governing pubertal onset and human reproduction, while alterations of KISSR are responsible for the development of hypogonadotropic hypogonadism syndrome that includes dysfunction of fertility, absent or incomplete sexual maturation, and puberty disorders. 3 4

Recently, clinical reports 5 6 7 8 described the ex novo insurgence of hypogonadotropic hypogonadism disorders such as precocious accelerated puberty, hypothalamic amenorrhea, and male hypogonadism, during the severe acute respiratory syndrome coronavirus 2 (SARS-CoV-2) pandemic. In spite of the importance of these clinical data, the issue has been overlooked 9 and, to the best of this author's knowledge, no molecular mechanism that might link the hypogonadotropic hypogonadism syndrome to the exposure to SARS-CoV-2 has been investigated and/or proposed.

Based on these observations, the present study posed a question: could SARS-CoV-2 infection/vaccination play a causal role via molecular mimicry and cross-reactivity in the diseases canonically ascribed to potential genetic variants of KISS1 and KISSR?

Consequently, molecular mimicry analyses were performed as follows. The amino acid (aa) sequences of KISS1 (Uniprot entry number: Q15726, 138 aa) and KISSR (Uniprot entry number: Q969F8, 398 aa) were retrieved from www.uniprot.org/ 10 and dissected into sequential pentapeptides offset by one residue (i.e., MNSLV, NSLVS, SLVSW, and so forth). The resulting pentapeptides were analyzed for occurrences in the SARS-CoV-2 proteome using the peptide match program (research.bioinformatics.udel.edu/peptidematch/index.jsp). 11 Human coronavirus 229E, Human respiratory syncytial virus B, and Mumps virus were utilized as controls. Pentapeptides were used as probes since a peptide formed by 5 aa residues defines a minimal immune determinant that can induce specific antibodies and specific antigen-antibody interaction. 12 13 14 15 The immunological potential of the peptide matching was analyzed by searching the Immune Epitope DataBase (IEDB, www.iedb.org/ ) 16 for SARS-CoV-2 immunoreactive epitopes hosting the shared pentapeptides.

The results of the molecular mimicry analyses are reported in Table 1 . As a first notable point, Table 1 shows that KISSR is the focus of an intense and specific peptide sharing with SARS-CoV-2. Numerically, 8 pentapeptides are common to the SARS-CoV-2 proteome and KISSR, while no sharing occurs with KISS1. In this regard, it has to be underscored that such a dimension of peptide sharing between SARS-CoV-2 and KISSR is unexpected and mathematically impossible. Indeed, assuming that all aa occur with the same frequency, the probability that one identical pentapeptide may occur in two proteins is 1 out of 20 5 (or 1 in 3,200,000 or 0.0000003125), that is, it is close to zero.

**Table 1:** Peptide sharing between SARS-CoV-2 and KISS1 and its receptor KISSR

Virus	Peptides a shared with:	
	KISS1	KISSR
Human coronavirus 229E (NCBI:txid11137)	–	CACYA
Human respiratory syncytial virus B (NCBI:txid79692)	–	–
Mumps virus (NCBI:txid11171	–	AAYAL
SARS-CoV-2 (NCBI:txid2697049)	–	ANLAA, AVVLL, LALHR, LFLVL, LRLGS, NLAAT, NPLLY, TVATS

a Peptides given in 1-letter code.

Then, the peptide commonality between SARS-CoV-2 and KISSR finds a logical scientific explanation in the close phenetic relationship between viruses and the origin of the eukaryotic cell. In fact, according to the endosymbiotic theory, 17 the first eukaryotic cell (our lineage) originated as a consortium consisting of an archaeal ancestor of the eukaryotic cytoplasm, a bacterial ancestor of mitochondria and a viral ancestor of the nucleus. Evolutionary, such a phenetic relationship, resulted in a sparse distribution of viral sequences in the human proteome. Immunologically, this means that targeting a viral protein inevitably leads to targeting human proteins, thus causing autoimmunity, 18

A second noteworthy point of the present study is the high immunological potential of peptide sharing. Indeed, exploration of IEDB revealed that all shared pentapeptides but one (namely, LRLGS) recur in 101 experimentally validated immunoreactive SARS-CoV-2-derived epitopes ( Table 2 ). That is, the potential immunologic cross-reactivity between SARS-CoV-2 and KISSR is high and powerfully suggests an autoimmune context for the hypogonadotropic hypogonadism disorders linked to KISSR alterations.

**Table 2:** SARS-CoV-2-derived epitopes containing peptide sequences common to KISSR

IEDB ID a	Epitope b c
1349	aflLFLVLi
4321	asANLAATk
26759	ikdlpkeiTVATSrt
37279	lLFLVLiml
48051	pkeiTVATSrtlsyy
48052	pkeiTVATSrtlsyykl
66952	TVATSrtlsy
100428	qliraaeirasANLAATk
531518	eiTVATSrtlsyykl
533455	rasANLAATkmsecv
1068860	aaeirasANLAATkm
1072541	sANLAATkmsecvlg
1074838	aeirasANLAATk
1074974	lLALHRsyl
1074999	mielslidfylcflaflLFLVLiml
1075003	NPLLYdanyflcw
1075083	TVATSrtlsyyk
1087755	tqqliraaeirasANLAA
1309418	aeirasANLAATkmsecvlg
1309534	nitrfqtlLALHRsyltpgd
1309938	rasANLAATkmsecvl
1310253	aeirasANLAATkms
1310513	itrfqtlLALHRsyl
1310529	keiTVATSrtlsyyk
1310547	kNPLLYdanyflcwh
1310592	lLALHRsyltpgdss
1310865	trfqtlLALHRsylt
1312358	eirasANLAATkmse
1312746	initrfqtlLALHRs
1312773	iraaeirasANLAAT
1313188	myasAVVLL
1313810	TVATSrtlsyyklga
1322562	NPLLYdany
1323750	rasANLAATk
1329417	fqtlLALHRsyltpg
1329597	iraaeirasANLAATk
1331140	crskNPLLY
1331247	dfylcflaflLFLVL
1332969	NPLLYdanyfl
1334248	vmyasAVVLL
1334326	yasAVVLLi
1334458	dikdlpkeiTVATSrt
1354273	ginitrfqtlLALHRsy
1377484	aghhlgrcdikdlpkeiTVATSrtls
1378052	cdikdlpkeiTVATSr
1382649	ikdlpkeiTVATSrtl
1383272	kdlpkeiTVATSrtls
1384629	lLALHRsyltpgdsss
1387524	rcdikdlpkeiTVATS
1392223	ikdlpkeiTVATSrtlsyyk
1394016	qtlLALHRsyltpgdss
1407859	aeirasANLAAT
1415369	cdikdlpkeiTVATS
1427956	eirasANLAATk
1464013	LALHRsyltpgd
1464014	LALHRsyltpgdsssgwt
1468599	lLALHRsyltpg
1496551	rasANLAATkms
1518333	trfqtlLALHRs
1539641	AVVLLilmtartvyd
1539752	crskNPLLYdanyfl
1539916	dlpkeiTVATSrtls
1541665	myasAVVLLilmtar
1542193	qtlLALHRsyltpgd
1542507	skNPLLYdanyflcw
1543352	wkcrskNPLLYdany
1584233	qliraaeirasANLAATkm
1596090	nitrfqtlLALHRsyltpgdsssgwtagaa
1596567	yvtqqliraaeirasANLAATkmsecvl
1597725	LALHRsyltpgdsssgwtagaaayy
1598225	aeirasANLAATkmsecv
1605379	ginitrfqtlLALHRsyl
1626811	pkeiTVATSrtlsyyk
1643627	aeirasANLAATkmse
1651464	dlpkeiTVATSrtlsy
1654327	eirasANLAATkmsec
1667866	irasANLAATkmsecv
1673173	LALHRsyltpgdsssg
1688275	qliraaeirasANLAA
1692097	sANLAATkmsecvlgq
1699067	tlLALHRsyltpgdss
1835518	trfqtlLALHRsyltpgdsss
1860045	tlLALHRsy
1865417	eirasANLAATkmsecvlgq
1866712	initrfqtlLALHRsyltpg
1870005	tqqliraaeirasANLAATk
1870081	TVATSrtlsyyklgasqrva
1871461	eirasANLAATkm
1873723	TVATSrtlsyykl
2001009	dikdlpkeiTVATSr
2001075	ginitrfqtlLALHR
2001123	irasANLAATkmsec
2001183	liraaeirasANLAA
2060884	rasANLAATkmsecvlgqsk
2116235	NLAATkmsecvlgqskrvdfcg
2116260	qtlLALHRsyltpgdsssgwta
2116290	tqqliraaeirasANLAATkms
2132218	LALHRsyltpgdsss
2133990	aeirasANLAA
2134243	qtlLALHRsyl
2135541	rfqtlLALHRsyltpgdsss

a Epitope IEDB IDs are listed in ascending numerical order. Details and references available at http://www.iedb.org/ .

b Epitope peptide sequences given in 1-letter code.

c Shared peptides given in capital letters.

## Conclusions

Starting from 2000, 19 this author's lab continuously reported that a massive peptide overlap exists between human and pathogen proteins, thus calling attention to the molecular mimicry and cross-reactivity issues in immunology and vaccinal protocols. 19 20 21 22 23 24 25 26 Here, this study describes the molecular mimicry and the immunologic cross-reactive potential between SARS-CoV-2 and KISSR, alterations of which are responsible for hypogonadotropic hypogonadism syndrome. 3 4

In essence, this study scientifically explains the clinical reports 5 6 7 8 on the onset of hypothalamic-pituitary dysfunctions following the SARS-CoV-2 pandemic and warrants further investigations, also in light of the scarce attention paid to the topic in relation to the emerging infectious disease outbreaks. 9 Clinically, the present data (1) lead to the inclusion of the hypogonadotropic hypogonadism syndrome among the SARS-CoV-2-related disorders that collectively form the coronavirus disease 2019 diseasome and (2) permit to catalog as autoimmune a syndrome until now defined idiopathic. 3 27 28
